# A Combined Study of Headspace Volatiles using Human Sensory, Mass Spectrometry and Chemometrics

**DOI:** 10.1038/s41598-020-64491-6

**Published:** 2020-05-08

**Authors:** K. G. McAdam, J. Tetteh, L. Bishop, H. Digard, J. Cote, S. Lubbe, C. Liu

**Affiliations:** 1 McAdam Scientific Ltd., 50 Leigh Road, Eastleigh, SO509DT UK; 2 DiKnow Ltd., 84 Rushdean Road, Rochester, Kent, ME2 2QB United Kingdom; 3 Research and Development, British American Tobacco Investments Ltd., Regents Park Road, Southampton, SO15 8TL UK; 4 Department of Statistical Sciences, University of Cape Town, Rondebosch, 7701 South Africa

**Keywords:** Mass spectrometry, Scientific data

## Abstract

Smokeless tobacco products (STPs) are widely used in certain parts of the world, yet there is limited understanding of how they are consumed, particularly the impact of chemosensory characteristics on their use. In order to develop an understanding of the drivers of STP use and product acceptability we conducted both human sensory panel testing and chemical analyses on a range of STPs. Free-sorting paired odour testing using sensory panellists identified similarities and clear differences between eleven different STPs. Headspace volatiles, analysed by headspace solid-phase microextraction gas chromatography mass spectrometry (HS-SPME-GC-MS), identified 20 to 70 components depending upon the STP. Key differences in headspace volatiles were found between STPs. For example, the headspace of Skoal Bandits Wintergreen was dominated by methyl salicylate, while Marlboro Spice consists of a more complex profile including pinene, nicotine, eugenol and cymene. Chemometric Target Factor Analysis (TFA) and Hierarchical Cluster Analysis (HCA) of chemistry and sensory data was used to deduce chemical drivers of sensory perceptions. The chemometric strategy used showed that headspace analysis is a complementary screening tool to sensory analysis in classification studies. This study is generic with applications across various product sectors that require routine human sensory panel evaluation.

## Introduction

The use of tobacco and nicotine consumption products are rapidly changing worldwide. In particular the use of e-cigarette and ‘Heat not Burn’ (HnB) devices are gaining popularity^1,2^. Use of such ‘Next Generation Products’ (NGPs) is related at least in part to the potentially lower risks of these products in comparison to cigarettes^3^, as the level of toxicants from NGPs is significantly lower^4–6^. One reason for lower toxicant emissions from these products is the absence of combustion (and associated high temperature toxicants) that occurs in a burning cigarette. Use of some low toxicant smokeless tobacco products (STPs) is also regarded as lower in risk than cigarette smoking^2,3,7,8^ although STPs are classified generically as International Agency for Research on Cancer (IARC) Group 1 Carcinogens^9^. Important distinctions exist between STPs and cigarettes in both the nature of the toxicants and the exposure sites in consumers. Cigarette use exposes the lungs of smokers to both tobacco-based toxicants and combustion products^10^. In contrast, STP use exposes the buccal-oral cavity, and to a lesser degree nasal and gastro-intestinal tissues, to tobacco-based rather than combustion-based toxicants^11^.

STPs encompass a variety of product types globally, with particular concentrations of use in Scandinavia, the USA and Indian sub-continent. In recent years use of the Swedish STP snus has grown in Norway^12^, Canada^13^ and South Africa^14^. There are significant differences in STPs compositions and manufacturing processes worldwide^15^. Two of the most widely used STPs in the USA and Europe are moist snuff and Snus respectively, both of which are composed of finely cut tobacco, high moisture levels (up to 60%), humectants, salt and flavour compounds [McAdam *et al*. 2019]. USA moist snuff is based on finely cut air-cured and fire-cured tobacco varieties that may be fermented^15^. Swedish snus consists of finely ground dark air-cured tobaccos that is pasteurized during manufacture to control constituent chemistry post-production; it is found as loose tobacco (loose snus) or portion form within a porous fleece material (portion snus).

The base component of STPs, tobacco, is a complex biomass system consisting of thousands of chemical constituents^16^ including cellulose/hemicellulose, lignin, pectin, sugars, proteins, amino acids, nicotine and related alkaloids. The chemical composition of STPs also diversify once manufacturing differences such as pasteurization and fermentation, additives, ageing and flavour compounds are factored in. Toxicant contents of STPs have received specific attention^11,17–25^. A review of STPs by the UK Royal College of Physicians noted that different health risks are associated with the use of different STPs in line with the levels of chemical toxicants within those products^7^.

As well as the contents of STPs, the way in which they are used also has important implications for individual and population risk. There is relatively little information available concerning the patterns and behaviours associated with STP use, in contrast to the information available for cigarette smoking^26,27^. Although studies have examined the durations and daily levels of snus consumption in Sweden^28^, and assessed the degree of STP constituent extraction during use^29^, relatively little is known about factors leading to STP use, product attribute preferences, or the identity and the sensory contributions of their chemical components^15^. Anecdotal evidence points to the importance of product aroma as an informal quality metric used by consumers on opening new packs of products, as a means of assessing product freshness. Many Swedish snus products have a traditional core product characteristic of an overtly ammoniacal-style aroma, modified by volatile top-flavours. In contrast, STPs from other countries have distinctly different general aroma characteristics reflecting national consumer preferences, with little ammoniacal character. In the USA moist snuff products flavourants such as wintergreen oil, spearmint, apple, cherry, vanilla are widely used^15^. These observations highlight levels of complexity in STP composition and consequent drivers of use that have received little attention to date, and these knowledge gaps are an important area to address.

In the field of Food Science, successful understanding of the interaction between consumers and the products they consume has been gained by combining human panel testing and chemical profiling^30,31^. Human panel testing enables analytically objective measurements to be made concerning product characteristics as perceived by human sensory organs^32^. Testing may be “descriptive”, with the aim of providing information on the influence of product parameters on sensorially perceptible properties; alternatively, “discriminatory” sensory analyses can define similarities or differences between products. Methods for discriminatory analyses include triangle tests, paired comparison, or free-sorting techniques. Use of trained panellists allows assessments to be conducted that are neutral and with minimised bias. The use of multiple subjects in panels, often between 8 and 20, increases the representativeness of the data generated. Human panel testing is a resource heavy activity, requiring substantial efforts in the creation, maintenance and use of an effective panel. Candidate panel members have to be screened for sensory acuity and commitment to panel testing; formal training to detailed procedures is required to ensure quality of data. Panellists are prone to fatigue, ageing, illness and lack of availability due to competing work priorities. Testing environments need to be free of distractions, with controlled lighting, ventilation and separation between subjects. Methodology needs to be robust to avoid bias, with random sample presentation, labelling and apparatus free from confounding odours. Given the resource requirements for effective panel testing, chemical analysis techniques are often sought as a lower-cost alternative.

However, chemical analysis of STPs, is challenging due to the diversity of tobacco constituents present^16,33^. Approaches that focus on the volatile components present in the headspace of smokeless tobaccos can overcome these problems through simplification of the analysed matrix. Headspace gas chromatography linked to mass spectrometry (HS-GC-MS) with or without solid-phase microextraction (SPME) is highly suited to volatile species analysis such as aldehydes, ketones, alcohols and esters, phenolic compounds and hydrocarbons^34^. These techniques are generally quite rapid, solvent-free, and require minimum sample preparation; they minimize analytical interference by extraction agents and co-extracted matrix constituents. HS-GC-MS has enabled the development of applications including qualitative fingerprinting and sample discrimination^35^, dynamic flavour monitoring and process optimization^36,37^, quantification of volatile flavouring compounds based on multivariate data modelling^38^ and high-throughput strategies in food and environmental analyses^39^.

A major challenge in HS-SPME/GC-MS methodology is to develop and use an effective data analysis strategy to accurately interpret the data, taking into consideration sample and chromatographic complexities. A typical chromatogram can yield many peaks, and the comparison of chromatograms across samples to identify key components that might influence sensory attributes can be a very challenging task^40,41^. The use of multivariate chemometric pattern recognition techniques has proved effective in dealing with such complex data sets. A review of chemometric pattern recognition in food analysis, including use of Principal Component Analysis (PCA), has been published^42^ and the technique has been successfully deployed in areas such as wine aging studies^43^.

Beyond use of pattern recognition for classification, there are situations where identification and quantification of components in complex mixtures are needed. In such situations self-modelling pattern recognition techniques such as Target Factor Analysis (TFA) has proven very effective in determining the relative quantities and identities of complex mixture samples without prior assumptions. The theory and applications of TFA have been detailed extensively elsewhere^44^. It is particularly suitable for resolving complex spectroscopic and chromatographic data^45,46^, for example monitoring drug permeation profiles through skin via infrared spectroscopy^47^. Besides TFA, use of clustering algorithm and visualization in the form of hierarchical dendrograms has proven very effective in classification studies. Spatial groupings can also be used to examine similarities between samples. Many reports have shown that such visualization techniques have been used in the monitoring of counterfeit drugs^48^, essential oils in botanic materials^49^ and gene expression for early immune response induced by live attenuated and inactivated influenza vaccines^50^. Theoretical details of hierarchical clustering dendrograms and applications of PCA techniques for classification can be found elsewhere^51^, but a brief description is provided here for clarity. Cluster analysis creates groups, or clusters of objects formed in such a way that objects in the same group share similar patterns or ‘features’ such as chemical components. The classification tree (dendrogram) is a multilevel hierarchy, where clusters at one level are joined together as clusters at the next level are formed. Similarities between objects are based on calculated (e.g. Euclidean) distances between samples. There are several clustering techniques and the choice depends on the data configuration and complexity^52^.

These approaches have been employed in a number of food science areas, ranging from olive oils to types of wines. These studies demonstrate that differences in sensory and flavour attributes between products are often linked to variations in their chemical compositions^40,53^. To date there has been little work on this aspect of product aroma and use ritual associated with STPs. Use of such chemosensory approaches with STPs is less well explored, but represent a promising approach to understanding smokeless tobacco product drivers of use. The current study is a first step towards building understanding in this area. We report a chemosensory analysis approach for discriminating STPs by identifying sensory differences and similarities between different smokeless products and explain such classifications on the basis of chemical composition. Our approach was to:

- identify difference and similarities between STPs, using free-sorting human sensory panel techniques,
- analyse core chemical constituents that characterise particular smokeless tobacco products, using Headspace-GC/MS,
- examine correlations between the human sensory data and analytical measurements using multivariate techniques, including principal component analysis (PCA), hierarchical clustering techniques and target factor analysis (TFA), to identify key chemical constituents of the STPs.

The studies involved flavoured and unflavoured snus as well as moist snuff products.

## Materials and Methods

In this section, we describe choice of STPs, sample preparation methods, the sensory panel methodology, the procedure for headspace volatile analysis and chemometric data analysis strategies.

### STPs

Table 1 lists the eleven smokeless tobacco products used in this study. One product (an unflavoured snus product) was deployed as two samples in the study, leading to twelve STP samples overall, plus a blank vial sample. STPs were chosen to provide diversity in terms of manufacturer, process, product type, country of sale, and presence/absence of flavour compounds as follows:

- Two products were unflavoured and flavoured variants of the same snus product sold in Sweden (samples 6 and 7 respectively), providing an examination of the importance of added flavour versus the base snus aroma/chemical profile.
- The unflavoured snus product was deployed twice in sensory studies as two separate products to provide a no-difference control check of the sensory panel performance.
- Swedish snus products from the same (Samples 7–9) and different manufacturer (Sample 10–11) highlighted discriminating power for products with similar general attributes
- Snus products from the same manufacturer sold within Canada, Norway, and Sweden (Samples 1, 2, 3 and 7–9) – each manufactured with the same tobacco but with country-specific flavour profiles, provides an examination of discriminating power with regards to STPs possessing very different flavour profiles
- Moist snuff products (Samples 4 and 5) with differing flavour descriptors, manufactured using very different approaches to the snus products in the sample set.

**Table 1:** List of products showing coding (abbreviation), market and style.

No.	Product Name	Code	Type	Market^a^	Style (Weight, g)^b^
1	du Maurier Original	DMO	Snus	Canada	Pouch (0.40)
2	Pall Mall Original Portion	PMOP	Snus	Norway	Pouch (1.00)
3	Pall Mall White Portion	PMWP	Snus	Norway	Pouch (0.80)
4	Marlboro Spice	MSS	Snus	USA	Pouch (0.23)
5	Skoal Bandits Wintergreen	SBWP	Moist snuff	USA	Pouch (0.70)
6	Lucky Strike Control	LSCnf	Snus	Sweden	Pouch (1.00)
7	Lucky Strike Original	LSOP	Snus	Sweden	Pouch (1.00)
8	Granit Loose	GraL	Snus	Sweden	Loose (0.50)
9	Granit Portion	GraP	Snus	Sweden	Pouch (1.00)
10	General Loose	GenL	Snus	Sweden	Loose (0.50)
11	General Portion	GenP	Snus	Sweden	Pouch (1.00)
12	Blank vials	Blk	—	—	—

^a^Market data in 2008. ^b^Maximum weight of sample used for headspace analysis is indicated in the brackets. Average weight of sample for sensory evaluation was 8 g.

Specifically, sample 1 (du Maurier Original) was a snus product sold in Canada. Samples 2 and 3 (Pall Mall Original and Pall Mall White Portion) were snus products available in Norway and samples 7–9 (Lucky Strike Original, Granit Loose, Granit Portion) were Swedish snus products. These products were obtained from the manufacturer Fiedler & Lundgren, in Sweden (now part of British American Tobacco). Sample 6 (Lucky Strike Control) is tobacco material comprising the Lucky Strike product as sold in Sweden in 2008, composed and manufactured as Sample 7 but without the addition of flavours. Samples 10 and 11 (General Loose, and General Portion) were Swedish snus products purchased from Swedish internet retailers. Samples 4 and 5 (Marlboro Spice, Skoal Bandits Wintergreen) were moist snuff products sold in the US in 2008, sourced from retail outlets in North Carolina. Abbreviated names in the table were used for graph labels.

### Sensory evaluation

Sensory tests (sniffing) were performed using in-house experienced sensory panelists. Tests were designed and conducted in accordance with Institute of Food Science & Technology Guidelines for Ethical and Professional Practices for the Sensory Analysis of Foods. Experimental designs were approved by the BAT in-house “Human Research Committee” review board. Subjects provided informed consent prior to commencement of studies.

Two sessions were conducted using a free-sorting approach[53, 54], resulting in two separate sets of scores. Sessions 1 and 2 involved 18 and 22 panelists respectively. Fourteen panelists took part in both sessions. There were no gender and age distinctions between the panels. The samples were grouped by panelists according to similarity in aroma.

Approximately 8 g of sample were placed in 3-digit coded 125 ml jars. Each panelist received all of the products stored in the jars at once. Jars were randomly arranged in a horizontal line (from left to right). The sniffing process is summarized as follows:

- Session 1: the panelists assessed each glass jar in the order from left to right. Panelists opened each jar, sniffed the content and replaced the lid. They were then asked to free-sort the jars into mutually exclusive groups based on similarities of aroma. The number of groupings for each panelist ranged from 2 to 11.
- Session 2: after a rest period of 20 minutes (during which panellists did not eat or drink), the panelists assessed the products in the same way as in session 1 to confirm or modify their initial groupings. The groupings after this stage were taken as the final results.

**Figure 1:**
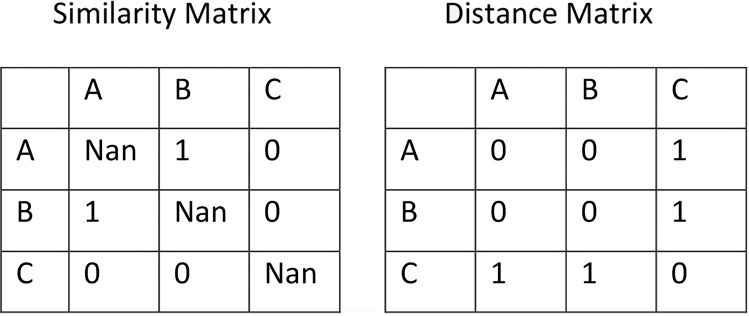
Similarity and dissimilarity matrices.

The scores were tabulated in a similarity data matrix with ‘0’ representing dissimilar and ‘1’ for similar odours. For example, Fig. 1 shows a similarity matrix with a sample population of A, B and C grouped as (A, B) and then C, where ‘Nan’ denotes ‘Not a Number’.

Similarities were converted to dissimilarity distance matrices^54^ by allocating 0 to the Nan diagonals, and assigning 1 to two products in different groups, Fig. 1.

**Figure 2:**
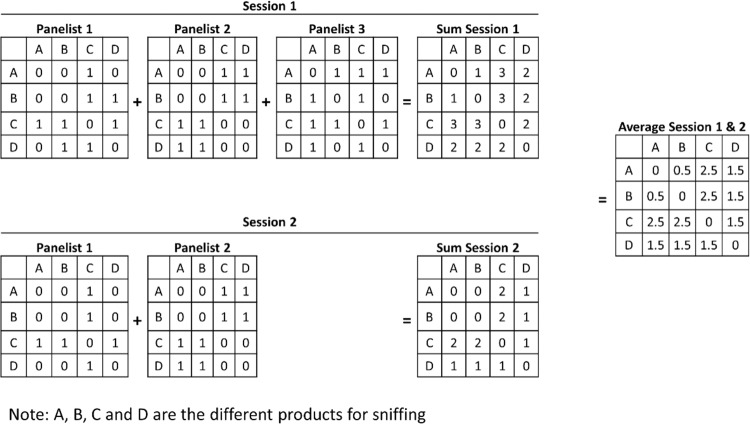
Schematic representation of the data collation process.

The distance matrix for all the panels in each session was summed across all products to yield a final score table for each session. An average distance matrix for the sessions was calculated. Cluster and spatial classification analysis were performed on averaged sensory data to enable direct comparison between the chemical and the human sensory data. Extended text on sensory characterization for consumer profiling has been published^55,56^. A schematic representation of the data collection process is presented in Fig. 2.

### Headspace extraction and analysis procedure

Headspace sampling of smokeless tobaccos used a 1 cm long (Supelco) SPME fiber coated with 100 µm polydimethylsiloxane (PDMS) phase. Sample weights are listed in Table 1. The differences in weights between products reflect average product weights per unit pouch in a container. The average weight of loose snus used was 0.5 g. Samples were placed in a 20 mL headspace vial. For semi-quantitative comparison of headspace compounds across the different samples, 50 µl of a solution of 37 µg/ml toluene-d8 internal standard in methanol (Sigma-Aldrich) was added to the samples. Each sample was incubated at 40 °C for 2 min. The SPME fiber was then inserted into the headspace above the sample which was also held at 40 °C for 2 min. The fiber was withdrawn and inserted into the split/splitless inlet of the GC instrument. The liner used was a 0.75 mm SPME (Supelco). The GC instrument used a 2 min desorption time at 280 °C. The fiber was then ‘baked out’ for 60 min at 280 °C to minimize carryover.

Duplicate HS-SPME/GC-MS analyses were performed on the majority of the samples. LSOP and DMO were run in triplicate. PMOP and PMWP were run once due to sample availability. Peak areas of the internal standard were monitored across different analyses as a QC measure. Relative peak intensity was compared by dividing the peak area by the toluene-d8 area.

### GC-MS instrumentation and conditions

An Agilent 6890 GC with an Agilent DB-WAX column (60 m x 0.25 mm ID x 0.25 μm film thickness, Agilent 5973 mass spectrometer and Gerstel MPS 2 autosampler was used for chemical profiling. Sample injection was in splitless mode with Helium carrier gas at a constant flow of 1 mL/min. The injector temperature was 280 °C. Initial oven temperature was 40 °C, held for 2 min, and ramped at 5 °C/min to 260 °C for 4 min. The MS was auto-tuned and operated in scan mode with no solvent delay with a 280 °C transfer line temperature, the quadruple mass selector temperature was 150 °C and the source temperature was 230 °C. The MS operated in EI mode at an ionisation energy potential of 70 eV, in full scan mode over a mass range of 29–400 amu.

### MS Peak Identification

Peaks were identified, where possible, using a flavour library from Agilent (Flavour2, containing approximately 1000 compounds) and also the Wiley library (version Wiley 7 N, containing approximately 500,000 compounds). To provide the best possible spectra for library matching, background subtraction was conducted to reduce signal noise, and co-eluting peaks were deconvoluted where necessary. Both of these steps were conducted using HP Agilent Chemstation. Library match factors less than 70% were denoted as tentative identifications. The identities of many of the flavour compounds used in this study were known to the project team. Where peak identification was regarded as tentative, samples of these compounds were analysed by matrix addition to establish retention times and confirm spectra in the analytical system. The use of both retention times and library matched mass spectra provided a 2-fold confirmation of identities across the whole data set. When measuring peak areas background subtraction was not used, and areas were quantified using ion extraction.

### Chemometric data analysis and visualization

For multivariate data analysis, a master GC-MS data table was compiled from all headspace chromatographic profiles. Rows of the master table contain the peak area ratio (to toluene-d8 internal standard) of all the identified chemical entities from 22 chromatograms. 205 unique chemical entities were identified resulting in a final data matrix of 205 compound rows (×) 22 columns of samples. A value of 0 was assigned when a compound was undetected. In practice, the peak identification from chromatograms carried varying degrees of uncertainty, which results from mainly unresolved peaks as well as human and software errors. The peak area ratio data table was analysed without any further pre-treatment or scaling.

#### Overview of Target Factor Analysis (TFA)

To determine the core chemical components that characterises each sample, the master GC-MS data table was subjected to TFA. Equations 1 to 4 summarises the main TFA process. The theory and applications of TFA have been detailed extensively elsewhere^44^.

1 $${\rm{D}}={\rm{RC}}+{\rm{E}}$$

2 $${{\rm{R}}}^{+}{{\rm{R}}}_{{\rm{t}}}={\rm{B}}$$

3 $${{\rm{B}}}^{+}{\rm{C}}={{\rm{C}}}_{{\rm{p}}}$$

4 $${\rm{RB}}={{\rm{R}}}_{{\rm{p}}}$$

**D** is the compiled 205-row GC-MS dataset (**R**) and 22 columns (**C**) of number of samples. **E** is the experimental error. **R** consists of the significant principal components (or factors) in row domain of **D**, and **C** consists of the significant principal components (or factors) in column domain of **D**. **B** is a transformation matrix deduced from the product of **R**^**+**^ and **R**_**t**_, **R**_**t**_ are the target vectors in the row domains of **D**. **R**^**+**^ and **B**^**+**^ are the generalized inverses of **R** and **B** respectively (i.e. **X**^**+**^ = [**X**^**T**^**X]**^**−1**^**X**^**T**^ and **X**^**T**^ is the transpose of **X)**. Several strategies have been reported for selecting **R**_**t**_. Ideally, a known set of pure vector profiles will be used but in practice this is not always possible. One approach is the use of partial vector profiles which are iteratively refined to form key set of unique factors. The approach is the so called needle search strategy (or partial target) described extensively elsewhere^47,57^. For example the value 1 is set at each of the rows of **R**_**t**_ keeping the rest at 0. These initial targets are refined and constraints such as non-negatively imposed. A selection of key vectors based on the refined target combinations spanning the significant factor space are then used to estimate **C**_**p**_ and **R**_**p**_. **C**_**p**_ and **R**_**p**_ are the estimated pure profiles in column and row domains of **D** to yield minimum noise or error. **D**_**new**_ = **R**_**p**_**C**_**p**._ The aim is to minimize the difference (**∆)** between **D** and **D**_**new**_ such that **|D-D**_**new**_ | **=∆**_**min**_. Here the core chemical components in **R**_**p**_ and corresponding sample intensities in **C**_**p**_ enables chemical uniqueness between samples to be deduced. A recent review highlighting these approaches and applications has been published^45^. Matlab® software^58^ was used for the analysis. The core programming codes were described previously^44^.

#### Overview of Hierarchical Cluster Analysis (HCA)

Euclidean distances based on the 205 by 22 data matrix of the chromatographic dataset were calculated across all samples and analyzed in the same way as the sensory data set. By convention, the cross product (similarity) matrix for both sensory and headspace data for each sample were calculated and used for the PCA^59–61^.

Dendrogram classification plots were generated based on average linkage and grouping methodology^15^. Other linkage methods explored in this study included complete, single, centroid and Ward. The number of clusters are obtained from visualization of the generated dendrogram layout. Extracted significant principal components were also used as a guide to deduce the number of clusters.

#### Application of Principal Component Analysis (PCA)

3-d scatter plots were used to visualize similarities between samples. This involved (a) conducting principal component analysis (PCA) (b) selecting the first n significant principal components as input for the number of clusters to be modelled (c) plot the identified clusters in x, y, z space. Using the PCA major variations in the data are captured in the main principal components (typically > 70% of the data). Use of principal components as input for classification reduces noise in the original data and highlights the core variations that characterizes the samples. In (b) Euclidean distances between each set of principal components were calculated and the relative closeness between each sample projected in x, y, z space. For each cluster, the distance from the cluster center to the edge was calculated and shaded in gray. The 3D scatter plot enables visualization of a collection of a number of principal components describing a sample projected into three-dimensional space. The Matlab® “clusterdata” and “scatter3” functions were used in this study^58^.

## Results and Discussion

The results and discussion are provided in three sections below. First, the results relating to the sensory assessment are presented. Subsequently the results of the general chemical classification based on the sensory distance matrix and headspace GC-MS data table are described. This is followed by results relating to TFA extraction of the core volatile chemical constituents associated with the sensory effects.

### Sensory assessment results

Table 2 presents the summed free-sorting distance matrices for the two sensory sessions described in methodology. Both sessions are shown together as (session 1, session 2). The data on the whole were consistent between the two sessions, with similar mean, median and mode scoring differences between the same product in different sessions of 4 panellists, irrespective of the magnitude of the difference between products. This facilitates power size calculations of optimum replicate numbers for future analyses of this kind.

**Table 2:** Free-sorting distance matrix data. (Session 1, Session 2).

Samples	LSCnf2	LSCnf1	PMWP	GraL	GenP	DMO	PMOP	LSOP	SBWP	GraP	GenL	MSS*
LSCnf2	0, 0	5, 8	14, 13	15, 22	15, 20	16, 16	9, 17	17, 19	17, 22	15, 22	16, 19	18, -
LSCnf1	5, 8	0, 0	16, 15	16,21	16,19	18, 19	12, 17	18, 18	18, 22	16, 22	16, 19	18, -
PMWP	14, 13	16, 15	0, 0	16, 20	14, 16	13, 13	12, 14	11, 17	18, 22	16,20	14,21	18, -
GraL	15, 22	16, 21	16, 20	0, 0	13, 18	17, 21	16, 20	14, 18	17, 20	10, 9	13,19	18, -
GenP	15, 20	16, 19	14, 16	13, 18	0, 0	15, 19	17, 18	9, 14	18, 22	15,16	10,19	17, -
DMO	16, 16	18, 19	13, 13	17, 21	15, 19	0, 0	14, 20	16, 19	18, 22	17,22	17,21	18, -
PMOP	9, 17	12, 17	12, 14	16, 20	17, 18	14, 20	0, 0	14, 15	18, 22	17,21	14,17	18, -
LSOP	17, 19	18, 18	11, 17	14, 18	9, 14	16, 19	14, 15	0, 0	18, 22	16,17	9, 17	18, -
SBWP	17, 22	18, 22	18, 22	17, 20	18, 22	18, 22	18, 22	18,22	0, 0	18,20	18,20	16, -
GraP	15, 22	16, 22	16, 20	10, 9	15, 16	17, 22	17, 21	16, 17	18, 20	0, 0	16,20	18, -
GenL	16, 19	16, 19	14, 21	13, 19	10, 19	17, 21	14, 17	9, 17	18, 20	16,20	0, 0	17, -
MSS*	18, -	18, -	18, -	18, -	17, -	18, -	18, -	18, -	16, -	18, -	17, -	0, -

Note: Layout shows sessions (1, 2.) e.g. LSCnf1 and LSCnf2 (5, 8) implies 5 for session 1 and 8 for session 2. MSS* (-) implies no sensory data for session 2.

Product Codes: Du Maurier Original (DMO), General Loose (GenL), General Portion (GenP), Granit Loose (GraL), Granit Portion (GraP), Lucky Strike Original (LSOP), Lucky Strike Control (LSCnt), Marlboro Spice (MSS), Pall Mall Original Portion (PMOP), Pall Mall White Portion (PMWP), Skoal Bandits Wintergreen (SBWP).

Table 3 shows the averaged sensory data from both sessions. In the table a relatively large value indicates strong dissimilarity between the samples. For example, an average of 20 panelists considered SBWP and LSOP dissimilar, whilst 9.5 identified dissimilarity between GraP and GraL. On average 6.5 panelists regarded the identical samples LSCnf1 and LSCnf2 as different, which provides an indication of the level of false positives, or ‘noise’ within the data. This error is lower than the smallest difference reported between different products (9.5).

**Table 3:** Averaged free-sorting distance matrix from sensory panels in Sessions 1, 2.

Average S1,S2	LSCnf2	LSCnf1	PMWP	GraL	GenP	DMO	PMOP	LSOP	SBWP	GraP	GenL	MSS
LSCnf2	0	6.5	13.5	18.5	17.5	16	13	18	19.5	18.5	17.5	18
LSCnf1	6.5	0	15.5	18.5	17.5	18.5	14.5	18	20	19	17.5	18
PMWP	13.5	15.5	0	18	15	13	13	14	20	18	17.5	18
GraL	18.5	18.5	18	0	15.5	19	18	16	18.5	9.5	16	18
GenP	17.5	17.5	15	15.5	0	17	17.5	11.5	20	15.5	14.5	17
DMO	16	18.5	13	19	17	0	17	17.5	20	19.5	19	18
PMOP	13	14.5	13	18	17.5	17	0	14.5	20	19	15.5	18
LSOP	18	18	14	16	11.5	17.5	14.5	0	20	16.5	13	18
SBWP	19.5	20	20	18.5	20	20	20	20	0	19	19	16
GraP	18.5	19	18	9.5	15.5	19.5	19	16.5	19	0	18	18
GenL	17.5	17.5	17.5	16	14.5	19	15.5	13	19	18	0	17
MSS	18	18	18	18	17	18	18	18	16	18	17	0

Product Codes: Du Maurier Original (DMO), General Loose (GenL), General Portion (GenP), Granit Loose (GraL), Granit Portion (GraP), Lucky Strike Original (LSOP), Lucky Strike Control (LSCnt), Marlboro Spice (MSS), Pall Mall Original Portion (PMOP), Pall Mall White Portion (PMWP), Skoal Bandits Wintergreen (SBWP).

**Figure 3:**
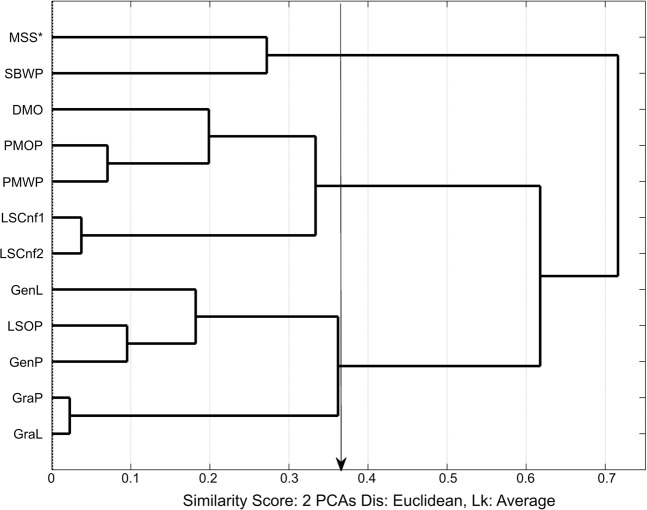
Hierarchical dendrogram plot from first two principal components for the averaged sensory data for sessions 1 and 2. Arrow shows similarity cutoff level at 0.36 units for three clusters. [MSS*, SBWP], [DMO, PMOP, PMWP, LSCnf(1,2)] and [GenL, LSOP, GenP, GraP, GraL]. Product Codes: Du Maurier Original (DMO), General Loose (GenL), General Portion (GenP), Granit Loose (GraL), Granit Portion (GraP), Lucky Strike Original (LSOP), Lucky Strike Control (LSCnt), Marlboro Spice (MSS), Pall Mall Original Portion (PMOP), Pall Mall White Portion (PMWP), Skoal Bandits Wintergreen (SBWP).

**Figure 4:**
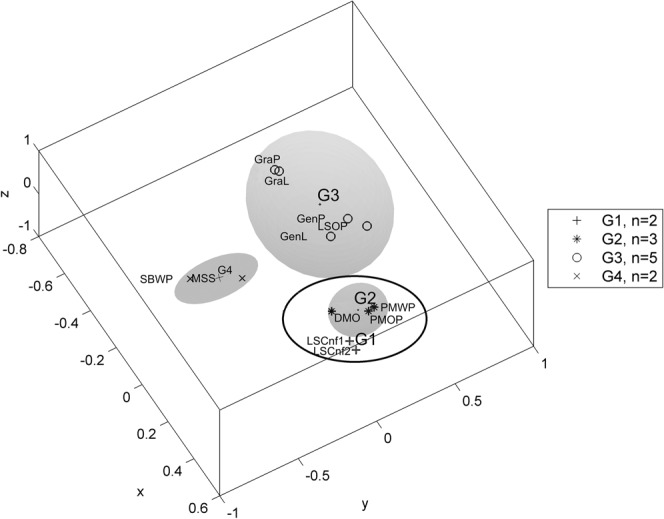
3D spatial classification from PCA and similarity grouping for averaged sensory sessions based on first two principal components (variance > 60%). The legend key shows four groups (G) and the number of products in each cluster. (G1 = LSCnf(1,2), G2 = PMOP, PMWP, DMO, G3 = GenP, GraL, GraP, GenL, LSOP. G4 = MSS, SBWP). Inserted circle shows the possibility of groups merging. Product Codes: Du Maurier Original (DMO), General Loose (GenL), General Portion (GenP), Granit Loose (GraL), Granit Portion (GraP), Lucky Strike Original (LSOP), Lucky Strike Control (LSCnt), Marlboro Spice (MSS), Pall Mall Original Portion (PMOP), Pall Mall White Portion (PMWP), Skoal Bandits Wintergreen (SBWP).

The averaged sensory data from both sessions (Table 3) were subjected to hierarchical and spatial cluster analysis. Figures 3 and 4 show the hierarchical dendrogram and 3D-scatter-PCA cluster plot respectively for the sensory classifications.

The hierarchical dendogram in Figure 3 is constructed from the first two principal components of the averaged data, and identifies sensory differentiation between products in-line with expectations based on known characteristics of the STPs. For example, the Swedish snus products Gra L and Gra P are very similar, consistent with their very similar composition; these products are different configurations (pouched and loose) of the same product. PMOP and PMWP were scored with a low level of dissimilarity by panelists. This closeness was expected as the two samples are already known to be very similar in tobacco and flavour composition, differing predominately by moisture levels with PMOP at 50% and PMWP at around 40% respectively. Using a Euclidian distance cut-off level of 0.3622 units, the dendrogram analysis grouped the 12 products into three sets, the group of Swedish snus products (Gen L, LSOP, Gen P, GraP and Gra L), the group of non-Swedish snus products (DMO, PMOP, PMWP and LSCnf(1,2)) and the two USA moist snuff products (MSS and SBWP).

In Figure 4, a 3D-spatial classification was constructed from PCA and similarity grouping for averaged sensory session data based on the first two principal components (Variance > 60%). The 3D-scatter-PCA cluster analyses identified the following groupings: G1 = [LSCnf(1,2)], i.e the two unflavoured snus products; G2 = [PMOP, PMWP, DMO], i.e. the three non-Swedish snus products; G3 = [GenP, GraL, GraP, GenL, LSOP], the five Swedish snus products; G4 = [MSS, SBWP], i.e the two USA moist products. The inserted circle around G1 and G2 shows the possibility of these groups merging, in agreement with groupings by the dendrogram analysis in Figure 3. Consequently, the sensory tests combined with data analysis were successful in correctly grouping and distinguishing products according to their flavour profile and STP type.

### General chemical classification

Table 4 shows the results obtained from the HS-SPME/GC-MS chromatogram of a du Maurier Original (DMO) snus sample. It shows a total of 36 identified peaks and one unidentified component marked “?”. The average numbers of peaks found in the other samples are 9 peaks for a Blank vial analysis, LSCnf had 23 peaks (“LSCnf(23)”), LSOP(61), GraL(56), GraP(66), GenL(54), GenP(63), PMOP(50), PMWP(42), SBWP(42), and MSS(71). The chromatograms are therefore complex.

**Table 4:** Peaks identified in the headspace of du Maurier Original (DMO) snus sample.

Peak	R.T.(min)	Compound	Area	Ratio to IS
1	3.16	Methanol	28322038	4.595
2	5.47	Toluene-d8	6164192	1.000
3	9.37	Limonene	564223	0.092
4	11.37	Cymene	28645	0.005
5	13.23	Methylheptenone	85526	0.014
6	14.43	Ethylhexylacetate	28358	0.005
7	16.41	Menthone + Acetic acid	112255	0.018
8	17.14	Ethylhexanol	104342	0.017
9	18.05	Benzaldehyde	581547	0.094
10	18.59	Linalool	52191	0.008
11	18.82	Linalylbutyrate	44665	0.007
12	19.14	Octadienone	25391	0.004
13	19.25	Acetoxypropanol	333179	0.054
14	19.68	Propylene glycol	1113860	0.181
15	20.28	Propanediol acetate	172959	0.028
16	22.28	Methylbenzyl acetate	102625	0.017
17	22.90	benzylacetate + β-Pinene?	281694	0.046
18	24.98	Acetylpyridine	46544	0.008
19	25.03	Anethole	33341	0.005
20	25.39	Benzaldehyde propylene glycol acetal	123223	0.020
21	25.49	Hexanoic acid	51022	0.008
22	25.66	Nicotine	16411055	2.662
23	26.09	Benzyl alcohol	10528049	1.708
24	26.79	Phenethyl alcohol	44658	0.007
25	26.96	Neophytadiene	563955	0.091
26	27.85	Cyclododecane	46868	0.008
27	31.74	?	28240	0.005
28	32.18	Myosmine	20582	0.003
29	32.32	Megastigmatrienone	44521	0.007
30	32.96	Piperonal	149511	0.024
31	33.43	γ-Decalactone	24609	0.004
32	34.10	Megastigmatrienone	26596	0.004
33	34.56	Nicotyrine	510219	0.083
34	34.87	Dihydroactinidiolide	38031	0.006
35	37.04	Dipyridyl	43698	0.007
36	37.67	Heliotropine propylene glycol acetal	164813	0.027
37	38.20	Isobutylphthalate	81474	0.013

**Figure 5:**
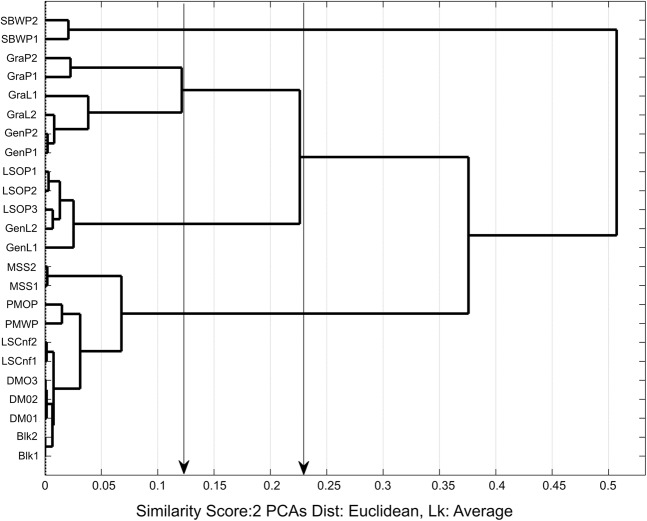
Hierarchical HS-SPME/GC-MS chemical classification profile based on first two principal components. The numbers behind the sample names indicate number of duplicate runs for each sample. Arrows show similarity cutoff levels around 0.12 and 0.22 units for 4 and 3 cluster analysis respectively. Product Codes: Du Maurier Original (DMO), General Loose (GenL), General Portion (GenP), Granit Loose (GraL), Granit Portion (GraP), Lucky Strike Original (LSOP), Lucky Strike Control (LSCnt), Marlboro Spice (MSS), Pall Mall Original Portion (PMOP), Pall Mall White Portion (PMWP), Skoal Bandits Wintergreen (SBWP).

Figure 5 presents the hierarchical dendrogram chemical classification profile based on the first two principal components, using the headspace chemical data from all twelve samples. Figure 5 shows that duplicate and triplicate samples are clustered similarly for all the samples. The groupings of similar samples confirm the reproducibility of the headspace chemical analysis. The two non-replicated samples, PMOP and PMWP showed a very close association as expected based on their compositions. The overall groupings in the samples were identifiable by examining the clusters at similarity levels at 0.22 and 0.12 on the x-axis in Figure 5 indicated by arrows. At about 0.22 similarity level, three clusters were clearly identifiable and at 0.12 four clusters were found.

The USA moist snuff sample SBWP, was found to be very different from the rest of the samples even in a two-cluster configuration. The difference between SBWP and the rest of the samples reflects the unique chemical profile of this product, as described below. With the three-cluster configuration (cut-off 0.22 units) the products were grouped as G1 = SBWP, G2 = the Swedish snus products (GraP, GraL, GenP, LSOP, GenL), G3 = the non-Swedish snus, unflavoured snus, blank sample and the USA MS product (MSS, PMOP, PMWP, LSCnf, DMO and Blk). A four-cluster configuration (cutoff 0.12 units) broke the Swedish snus products G2 of the three-cluster analysis into (GraP, GraL, GenP) and (LSOP, Gen L). Only by moving to a lower Euclidean distance could a 5th group be identified, in which the moist snuff product MSS was distinguished from the non-Swedish snus products, unflavoured snus, and blank.

**Figure 6:**
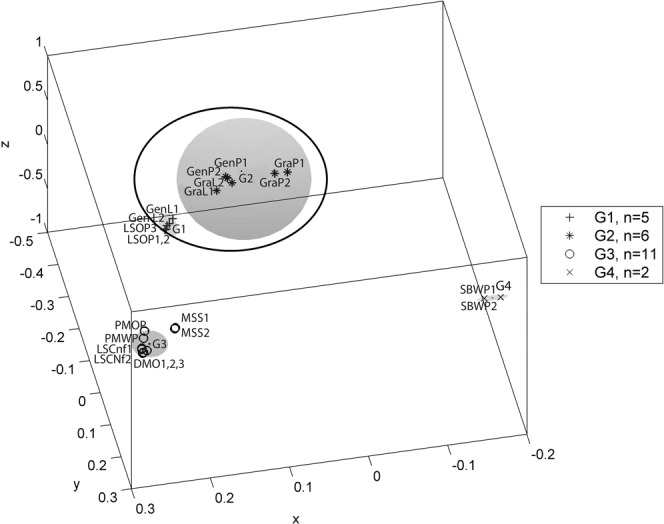
3D spatial classification from first two principal component analysis (>80% variance) and similarity grouping for HS-SPME-GC-MS. G1-G4 are the number of sample (n) in each group. The shaded zone highlights each cluster and scope. (G1 = GenL(1,2), LSOP(1,2,3). G2 = GenP(1,2), GraL(1,2), GraP(1,2). G3 = PMOP, PMWP, LSCnf(1,2), DMO(1,2,3), Blk(1,2), G4 = SBWP(1,2). Inserted circle shows the possibility of groups G1 and G2 merging. Product Codes: Du Maurier Original (DMO), General Loose (GenL), General Portion (GenP), Granit Loose (GraL), Granit Portion (GraP), Lucky Strike Original (LSOP), Lucky Strike Control (LSCnt), Marlboro Spice (MSS), Pall Mall Original Portion (PMOP), Pall Mall White Portion (PMWP), Skoal Bandits Wintergreen (SBWP).

Figure 6 shows a 3D spatial classification of the same headspace data used to generate Figure 5. The plot was based on the first two principal components (>80% variance) as input for the cluster analysis with a 4-cluster configuration based on groupings identified in Figure 5. The total numbers of samples (n) in each group (G) for the plot are shown in the figure legend. The groupings in Figure 6 are G1 = one set of Swedish snus samples: GenL(1,2), LSOP(1,2,3). G2 = a second set of Swedish snus samples: GenP(1,2), GraL(1,2), GraP(1,2). G3 = all other STPs except SBWP: i.e. PMOP, PMWP, MSS(1,2), LSCnf(1,2), DMO(1,2,3), Blk(1,2) and G4 = SBWP(1,2). The shaded area shows the approximate scope of each cluster from the center to the furthest sample in the cluster. The inserted ellipse shows the possibility of merging G1 and G2 (all of the Swedish snus samples) after visualisation.

Examination of the two graphs in Figures 5 and 6 shows that the visualization methods (i.e. Hierarchical Dendrogram and 3D-scatter-PCA plots) produced similar groupings for either 3 or 4 cluster models. The identified clusters are summarized in Table 5. The grouping of known replicated headspace analysis in each cluster confirms the consistency in the GC-MS data set. The results also show the flexibility of adopting one or both data treatment and visualization techniques for classification of this type of data. The results in Figures 5 and 6 shows at least 3 consistent clusters distinguished in Table 5 (note the use of **bold**, normal and *italics* fonts to distinguish groups) ‘**bold**’ (**DMO, MSS, PMOP, PMWP, LSCnf)**, ‘normal’ (LSOP, GenL GraP, GraL,GenP) and ‘italic’ (*SBWP*), fonts. Note that **MSS** was bold as well as italic in some cases and reflects some uncertainty in placing it in either the bold or italic group.

**Table 5:** Comparison of product groupings identified from sensory and headspace SPME-GC-MS dendogram and 3D-scatter-PCA analysis approaches.

Headspace SPME-GC-MS dendogram groupings based on Figure 5.	
Similarity Level=0.2263 (3 Clusters)	Similarity Level=0.1215 (4 Clusters)
LSOP, GenL, GraP, GraL, GenP	[LSOP], [GenL]
	[GraP], [GraL], [GenP]
*SBWP*	*SBWP*
**DMO, MSS, PMOP, PMWP, LSCnf**	**DMO, MSS, PMOP, PMWP, LSCnf**
**Sensory Dendrogram groupings based on** **Fig**. **3**.	
Similarity Level=0.36 (3 Clusters)	Similarity Level=0.36* (4 Clusters)
LSOP, GenL, GraP, GraL, GenP	LSOP, GenL, GraP, GraL, GenP
	*[MSS]*
*MSS, SBWP*	*[SBWP]*
**DMO, PMOP, PMWP, LSCnf**	**DMO, PMOP, PMWP, LSCnf**
**Headspace SPME-GC-MS 3D-scatter-PCA grouping based on** **Fig**. **6**.	
3 Clusters	4 Clusters
LSOP, GenL GraP, GraL, GenP	[LSOP], [GenL]
	[GraP], [GraL], [GenP]
*SBWP*	*SBWP*
**DMO, MSS**, **PMOP,PMWP, LSCnf**	**DMO**, **MSS**, **PMOP, PMWP, LSCnf**,
**Sensory 3D-scatter-PCA grouping based on** **Fig**. **4**.	
3 Clusters	4 Clusters
LSOP, GenL, GraP, GraL, GenP	LSOP, GenL, GraP, GraL, GenP
*MSS, SBWP*	*MSS, SBWP*
	**[LSCnf]**
**DMO, PMOP, PMWP, LSCnf**	**||DMO** | | , [**PMOP], [PMWP]**,

Note: X* indicates visual regrouping at the same similarity level. [X] indicates samples separated from original clusters in the smaller cluster analysis; ||X | | indicates possibility of relocating X from the current cluster. Identified clusters are distinguished by ‘bold’, ‘normal’ and ‘italic’ fonts. Product Codes: Du Maurier Original (DMO), General Loose (GenL), General Portion (GenP), Granit Loose (GraL), Granit Portion (GraP), Lucky Strike Original (LSOP), Lucky Strike Control (LSCnt), Marlboro Spice (MSS), Pall Mall Original Portion (PMOP), Pall Mall White Portion (PMWP), Skoal Bandits Wintergreen (SBWP).

It can be observed that in both representations the two SBWP samples showed distinct attributes in comparison to the rest of the samples. They were correctly grouped together by the headspace analysis in the two graphical representations. The US Marlboro Spice (MSS) Snus Pouch was found to exhibit a distinct sub-cluster within the ‘bold’ group of samples. It was noted that apart from the MSS sample, all the samples in bold are non-Swedish snus products from Canada, USA, and Norway. It was noted that the normal font group is the commercial snus products from Sweden. It was further noted that the flavoured Lucky Strike Original and unflavoured Lucky Strike Control from Sweden were clustered in different groups and this reflects the strong determinants of flavours in the grouping.

The results show that a clear grouping based on headspace data has been achieved. Distinctions between product groups was slightly less of a precise match to the general product styles than found with the sensory analysis, particularly in respect of the headspace grouping of the moist snuff product MSS with the non-Swedish snus products. This may reflect the influence of non-sensorially relevant headspace compounds in the chemical analysis that differed between samples without offering distinctive contributions to the headspace aroma.

### Specific chemical profiling

**Figure 7:**
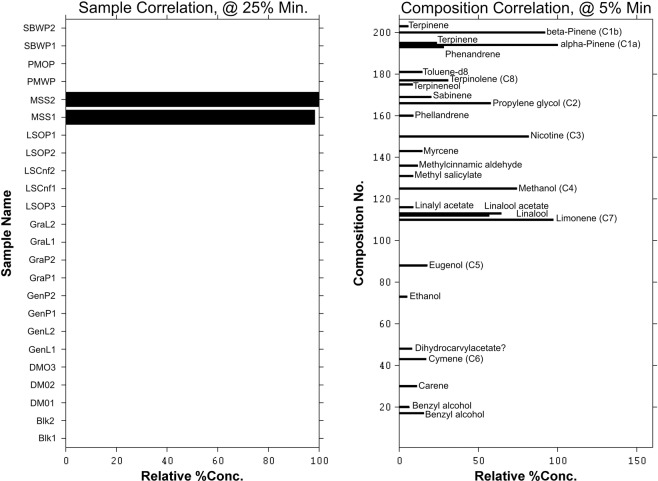
Multivariate target factor analysis output for HS-SPME/GC-MS data showing one of the factors based on six principal component model. The bar graphs on the left show the samples (MSS) identified and associated core components on the right. Some of the main chemical entities are highlighted; C1 = pinene, C2 = propylene glycol, C3 = nicotine, C4 methanol, C5 = eugenol and C6 = cymene.

**Figure 8:**
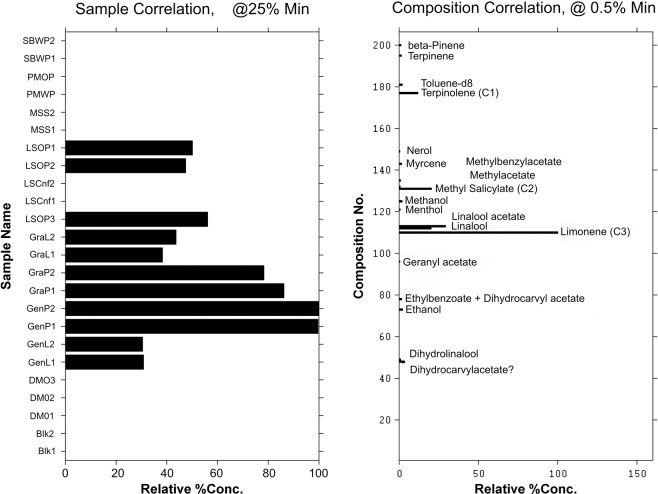
Multivariate target factor analysis output for HS-SPME/GC-MS data showing one of the factors based on six principal component model. The bar graphs on the left showing a combination of samples identified (LSOP, GraL, GraP, GenP and GenL) and associated core components on the right. Some of the main chemical entities are highlighted; C1 = terpinolene, C2 = methyl salicylate, C3 = limonene. Product Codes: Du Maurier Original (DMO), General Loose (GenL), General Portion (GenP), Granit Loose (GraL), Granit Portion (GraP), Lucky Strike Original (LSOP), Lucky Strike Control (LSCnt), Marlboro Spice (MSS), Pall Mall Original Portion (PMOP), Pall Mall White Portion (PMWP), Skoal Bandits Wintergreen (SBWP).

**Figure 9:**
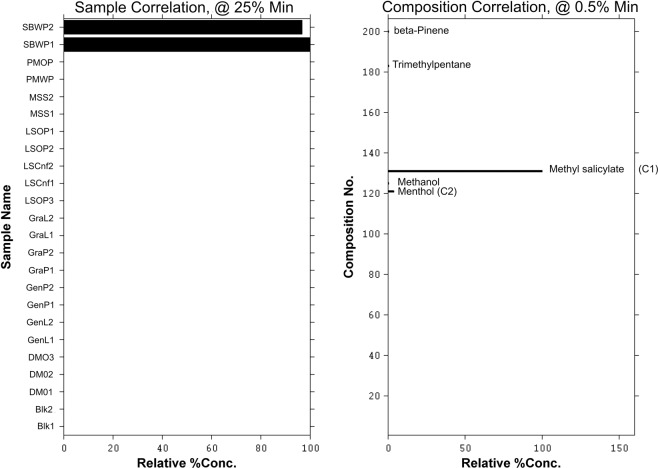
Multivariate target factor analysis output for HS-SPME/GC-MS data showing one of the factors based on six principal component model. The bar graphs on the left show the samples (Skoal Bandits Wintergreen - SBWP) identified and associated core components on the right. Some of the main chemical entities are highlighted; C1 = methyl salicylate, C2 = menthol.

Although it was very useful to group the samples based on the headspace volatiles, there is no specific chemical information in the results presented so far. An insight into the core chemical constituents should provide a further understanding of the sample characteristics. It is however a challenging task to visually examine all 22 chromatograms and accurately determine the constituents that characterizes uniqueness of a sample. The core chemical entities are to be selected from 205 rows of chromatographic data points. The multivariate Target Factor Analysis (TFA) approach described earlier was used to analyse the data. Figures 7–9 show results for the multivariate TFA output. The bar graphs on the left of each figure (representing transformed PCA loadings) show the samples identified and their associated components are shown on the right of each figure (representing transformed PCA scores). The y-axis of the graphs on the right shows the index number for the 205 compounds found in the headspace mass spectral profiles across all the samples analyzed. The intensities of the score for the chemical components were normalized to between 0–100. Where the relative concentration is below a set minimum percentage, the composition score was set to 0 to enable clarity of visualisation. Components identified above the set minimum threshold are labelled with the sample names in the bar graph on the right. Six principal components were used to model the TFA model. The profiles for three of the six TFA groupings are presented in Figures 7–9.

In Figure 7, the two Marlboro Spice (MSS) replicate analyses in the left-hand graph show that these two chemical profiles are indeed similar and distinct from the rest of the samples. No other product registered a presence at greater than 25% relative loadings threshold limit in the left-hand bar graph. This result shows that the rest of the samples do not share this unique chemical profile shown in the right-hand bar graph. Some of the components identified that could influence the perceived headspace aroma of MSS include alpha and beta pinene, propylene glycol, nicotine, methanol, eugenol, cymene, limonene and terpinolene. These components are marked C1a,b, C2, C3, C4, C5, C6 and C8 respectively in the right-hand graph. The common aroma characteristics of the key compounds are provided in the Supplemental Information. Of these compounds alpha and beta pinene, eugenol, cymene and limonene are recognized commonly used flavor compounds, and they contribute pine-like, wood-green, spicy clove and citrus notes to the headspace. In contrast, propylene glycol, nicotine and methanol offer less obvious contributions to the overall headspace aroma. This reflects a general weakness of chemical analysis approaches in relation to sensory analyses, wherein GC-MS analyses compounds of low sensory contribution are registered with equal weighting as those with high sensory impact. Several other trace chemical entities are also visible in Figure 7, and they clearly highlight the complex nature of the headspace components for this product.

Figure 8 shows a group of samples consisting of the Swedish commercial snus products LSOP, GraL, GraP, GenP and GenL that share common headspace characteristics. The heights of the bars in the left-hand bar graphs indicate the degree of significance for each sample relative to the identified chemical components in the right-hand bar plot. The highest response samples in the group were identified as GenP1 and GenP2 followed by GraP1 and GraP2. They all share the volatile profiles in the right-hand graph with varying intensities. Some of the constituents contributing to this group include limonene (the highest contribution, C3), methyl salicylate (C2) and terpinolene (C1). Aroma characteristics of these compounds are provided in the Supplemental Information. The three compounds provide herbal, wintergreen and citrus elements to the headspace. Again, several other components are identifiable but only in trace levels and they include linalool acetate and geranyl acetate. Figure 9 shows the moist snuff SBWP profile. This product is dominated by methyl salicylate (C1) and menthol (C2); the aroma characteristics of which are summarised in the Supplemental Information. The predominant aroma character is a wintergreen peppermint character. Traces of methanol, beta-pinene and trimethylpentane were also found to be minor contributors. The chemical composition complexity shown in Figure 7 clearly contrasts that shown in Figures 8 and 9 and highlights the effectiveness of using multivariate chemometric approach to analyze and determine the components that may be influencing the observed groupings.

### Relating sensory analysis to chemical profiles

Human olfactory responses may not necessarily correlate directly with the presence and quantity of chemical species in a sample as this is a complex phenomenon. Detailed study of olfactory responses to aroma may be found in the literature^41^. Aroma intensity is related to molecular structure, physicochemical properties and combinations of chemical mixtures. The relative intensities of components identified in this study and linked to each sample will therefore need further investigation. From sensory perspectives, compounds with strong aroma may be present in lower relative concentrations and may be missed in the headspace data analysis. Therefore, the headspace chemical classifications presented so far do not automatically suggest that the identified chemical compounds are the only entities that may be influencing the human sensory perception. In addition, as described above, some compounds differentiating the detailed headspace chemical profiles of these samples are of low sensory impact, and these can bear strongly upon groupings and differentiation between products. It is therefore important to examine the general headspace profiles and to deduce correlation with data from the panel of sensory judges. This type of correlation should provide some initial insight relating to chemistry and aroma of the products studied.

To enable direct comparison between headspace and sensory data, the averaged sensory data from both sessions (Table 3) and the HS-SPME/GC-MS data were subjected to the same hierarchical and spatial cluster analysis. Visual examination of the results shows that generally, headspace cluster plots have tighter groupings than the sensory clusters. This is an indication of confidence or level of uncertainty in the sensory data. To enable direct comparison between the HS-SPME/GC-MS and the sensory results, Table 5 provides a direct comparison of both dendrograms and 3D-scatter-PCA plot analyses.

The dendogram comparison in Table 5 shows clusters at 0.22 and 0.12 for the headspace and 0.36 and 0.36* similarity levels (x-axis) for the sensory results respectively. These similarity levels generated 3 and 4 clusters. In the tables, ‘X*’ indicates visual relocation of clusters for the similarity level at ‘X’ and the relocated sample(s) are in square bracket ‘[]’. Note that | |X | | indicates that X could be relocated after visual examination. The tables were evaluated by comparing similar cluster groups. For example, in the dendrogram analysis, the groups in the column heading “Similarity Level = 0.2263”, (Dendrogram Cluster 3, Figure 5) for headspace SPME-GC-MS results were compared with column heading “Similarity Level = 0.36” (Dendrogram Cluster 3, Figure 3) for sensory results. Table 5 shows the comparison between the “HS-3D-scatter, 3 Clusters” and “Sensory-3D-scatter, 3 Clusters” for headspace and sensory respectively.

The tabulated results show at least three distinct groups. The different groups are assigned different fonts; “**bold”**, “normal” and “italics**”**. They consist of (**DMO, MSS, PMOP, PMWP, LSCnf**), (LSOP, GenL GraP, GraL,GenP) and (*SWPB)*. The same three main groups were observed for both sensory and headspace results in the dendrogram and 3D-scatter-PCA plots. These consistent results are important and suggest that the analytical approach in this study has the capability to discriminate accurately between the sample based on headspace volatiles or sensory classifications. The results also show that headspace volatiles could be correlated with sensory data depending on the types of samples. Within the three main groups identified, sub-clusters were noted in the sensory as well as the headspace data. For example, in Table 5, the 3-cluster configuration isolated SBWP from the rest of the samples in the headspace analysis but in the sensory analysis SBWP was linked to MSS. In the 4-cluster configuration the ‘normal’ font group was split into [LSOP, GenL] and [GraP, GraL GenP] but there was no splitting in the sensory analysis. The sub-clusters in the headspace showed discriminatory ability in this data. In the 4-cluster configuration however, for the dendrogram, sensory analysis discriminated between MSS and SBWP but not in headspace analysis.

## Summary of Results and Discussion

The observed analytical agreement between the two classification configurations (i.e. 3 and 4 clusters) in the dendrogram and the 3D-scatter-PCA plots show that the two techniques complement each other. It is not possible by these results alone to suggest that the dendrogram visualization approach is better than the 3D-scatter-PCA plots in this study. It is however worth noting that the 3D-scatter-PCA plots have the option for the analyst to interact with the results by rotating clusters to see various perspectives of groups and this could be advantageous. The chemometric TFA approach enabled the determination of core chemical components associated with each sample. The identified components could be major contributors to the observed classifications by human panelist. For example, in Figure 8, the dominating chemical entities that linked the group of products together in the ‘normal’ font group are easily identified (Supplementary Figure S1). There appears to be a small contribution in the analysis of chemical headspace profiles from non-sensorily relevant compounds, and the impact of these species on the relationship with sensorial profiling is a topic that merits future exploration.

### Consistency of the method

During the study some assessment was made of the method repeatability. Short-term consistency assessment is of value in identifying the potential for errors in links between chemical and sensory testing. Reproducibility is viewed as being of lesser relevance for this technique, as the two primary applications of the approach (identification of sensorily relevant flavour compounds, and replacement of sensory panels with inherently less resource-intensive analytical techniques) are not repeated measures approaches requiring repetitive testing in multiple locations. Two main aspects of repeatability were considered, analytical and sensory, while product consistency was also briefly considered.

- Analytical repeatability:
  During the analytical method validation a repeatability exercise was conducted to establish the level of consistency between replicate analyses (as measured by the relative standard deviation). The same operator, product mix and testing equipment was used for this exercise. A Swedish snus product test-piece was prepared by loading an unflavoured snus tobacco base with a model mixture of 10 product-relevant flavours. Samples were run through the analytical method ten times, and peak areas compared. The data is shown in Supplementary Table S1. The coefficients of variation (C of V) for all of the flavours, and the internal standard, were below 10%. The C of V for nicotine (arising from the tobacco blend) was slightly higher at 11.6%. This latter value may provide some indication of the variability of the tobacco blend in the snus test-piece. However, the low levels of variability in the data were viewed as offering acceptable levels of performance for the application.
- Repeatability of the sensory panel.
  During the sensory assessment exercise panel members assessed the products twice, in separate sessions separated by a short time interval. To gain insights into repeatability of the free-scoring technique we compared the magnitude of the scores from both sessions (Table 2) for each product comparison. The analysis showed (Supplementary Figure S2) that the scores from the two sessions were strongly correlated (p < 0.001). There were, however, suggestions of a bias towards higher scores in the second session. The levels of variance between the two sessions were greater than seen in the analytical technique, confirming that sensory assessments are intrinsically more open to variation than controlled instrumental methods.
- Product Variability:
  The impact of product variability on links between analytical and sensory approaches was not assessed in this study, as it was not relevant to the intended applications. However, it should be noted that modern smokeless tobacco product manufacturing methods are designed to provide consistent products over time, particularly with respect to flavour character. The combination of quality measures in the flavour supply chain, use of synthetic flavours, modern dosing equipment, QC testing and statistical process control have reduced product and batch-to-batch variability significantly in recent years.

## Conclusions

The results obtained have shown that complex data from headspace GC-MS and sensory evaluations of smokeless tobaccos can be combined and analyzed using chemometrics to derive insights into the flavour characteristics of STPs. Little is currently understood about the chemical profiles of smokeless products headspace aromas. However, the results obtained in this work demonstrate that paired free-sorting sensory evaluation approaches combined with analytical techniques such as HS-SPME/GC-MS and chemometric techniques can be applied in a coherent manner to conduct detailed investigations of smokeless tobacco products. The results obtained provide valuable information with respect to chemistry and sensory differences. In particular, similarities and differences observed in the product classifications have provided a means of identifying sensorily relevant chemical species in these tobacco products. This information is of great interest to scientists seeking to understand the appeal and attractiveness of smokeless tobacco products.

The results also show that HS-SPME/GC-MS may be used as a screening protocol where fast chemical and sensory classification is required, such as in product development exercises, product shelf-life studies and, flavour substitution exercises. This approach can help to minimize or avoid unnecessary human involvement particularly at early screening stages of product surveys. Depending upon the scale of the product differences under investigation further work may be required to establish necessary levels of resolving power between similar product types.

The results further demonstrate the value of multivariate chemometric data processing for data of this kind. Without the use of multivariate chemometric techniques such as TFA, the identification of core chemical components from the complex chromatograms generated from the headspace analysis would be very cumbersome and challenging. For example, the core chemical constituents identified out of 42 peaks that correlated with the sensory classification for Marlboro Spice (MSS) are alpha and beta-pinene, propylene glycol, nicotine, methanol, eugenol and cymene. For Skoal Bandits Wintergreen (SBWP) the identified core components out of 71 are methyl salicylate, and menthol, trimethylpentane and beta-pinene. The analytical process presented in this report is generic and can be applied to other areas requiring similar insights.

## Supplementary information

Supplementary Information.
