# Supplementary material for: A Combined Study of Headspace Volatiles using Human Sensory, Mass Spectrometry and Chemometrics
Source: Sci Rep. 2020 May 8;10:7773. doi: 10.1038/s41598-020-64491-6 (PMC7210946; doi:10.1038/s41598-020-64491-6)

**A Combined Study of Headspace Volatiles using Human Sensory, Mass Spectrometry and Chemometrics**

K.G. McAdam, J. Tetteh, L. Bishop, H. Digard, J. Cote, and S. Lubbe and C. Liu

Supplementary Information

**Supplementary Table S1:**

Repeatability measurements of the analytical method applied to a smokeless tobacco product loaded with a model mixture of product relevant flavours.

Supplementary Figure S1:

Identities of the flavour compounds identified as showing differentiation between smokeless tobacco products aroma.

Supplementary Figure S2: Regression analysis to compare scoring differences between 1^st^ and 2^nd^ assessment panel sessions.


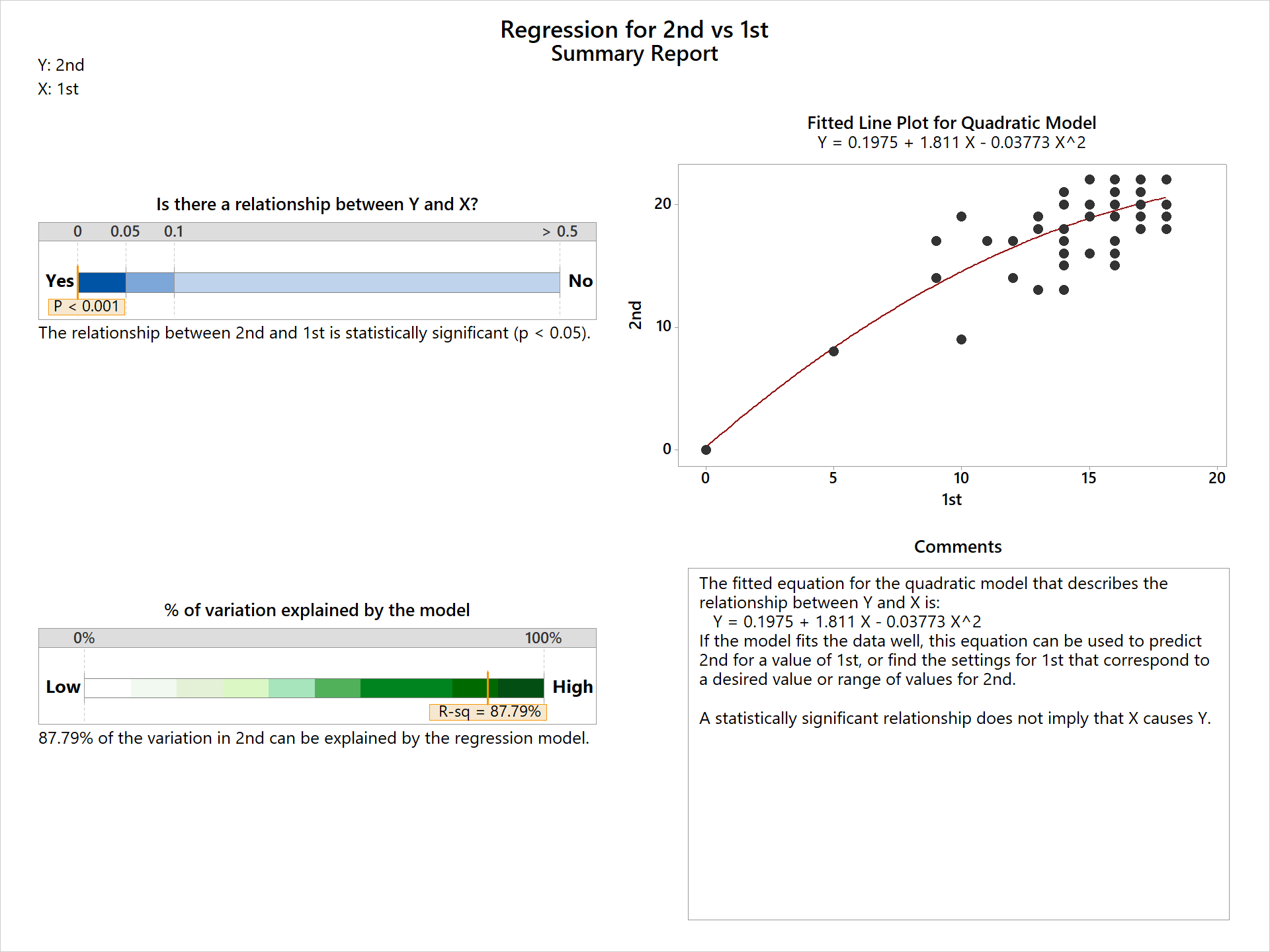

Supplement: Supplementary file 1 — Supplementary Information. [file 41598_2020_64491_MOESM1_ESM.docx]
